# Prevalence of post-traumatic stress disorder, acute stress disorder and depression following violence related injury treated at the emergency department: a systematic review

**DOI:** 10.1186/s12888-018-1890-9

**Published:** 2018-09-25

**Authors:** Robbin H. Ophuis, Branko F. Olij, Suzanne Polinder, Juanita A. Haagsma

**Affiliations:** Department of Public Health, Erasmus University Medical Center, PO Box 2040, 3000 CA Rotterdam, The Netherlands

**Keywords:** Depression, Post-traumatic stress disorder, Trauma, Violence, Injury, Emergency department, Prevalence

## Abstract

**Background:**

In order to gain insight into the health impact of violence related injury, the psychological consequences should be taken into account. There has been uncertainty regarding the prevalence of posttraumatic stress disorder (PTSD), acute stress disorder (ASD), and depression among patients with violence related injury. An overview of prevalence rates may inform our understanding of both prognosis and recovery for these patients. Therefore, we aim to provide an overview of the published literature reporting the prevalence rates and trajectories of PTSD, ASD, and depression following violence related injury, and to assess the quality of the studies included.

**Methods:**

A systematic review was conducted in order to provide an overview of the published literature reporting the prevalence of PTSD, ASD and depression following violence related injury treated at the emergency department or hospital. The EMBASE, MEDLINE, Cochrane Central, PubMed, and PsycINFO databases were searched systematically. The quality of the included studies was assessed.

**Results:**

We included sixteen studies reporting the prevalence rates of PTSD, ASD, or depression. Clear prevalence trajectories could not be identified because the range of prevalence rates was diverse at each time point. Heterogeneity resulting from the use of different diagnostic instruments limited comparability. The included studies were susceptible to bias due to low response rates and loss to follow-up.

**Conclusions:**

The differences in diagnostic instruments limited comparability of the prevalence rates. Therefore, clear prevalence trajectories could not be identified. Study participation and loss to follow-up require more attention in future studies. Uniformity in diagnostic procedures is needed in order to draw general conclusions on the prevalence of PTSD, ASD, and depression following violence related injury.

**Electronic supplementary material:**

The online version of this article (10.1186/s12888-018-1890-9) contains supplementary material, which is available to authorized users.

## Background

More than 1.5 million people worldwide die from violence related injury every year, and even more people suffer from non-fatal injury caused by violence [1]. Approximately 1.4 million non-fatal violence related injuries are treated annually in hospital emergency departments (ED) in the US [2]. In Western Europe, 670,000 violence related injuries require medical treatment each year [3]. According to the diagnostic and statistical manual for mental disorders (DSM), exposure to serious injury is an example of a traumatic event [4]. Longitudinal studies of responses to traumatic events show that mental disorders such as post-traumatic stress disorder (PTSD), acute stress disorder (ASD), and depression frequently occur after experiencing a traumatic event, although the course can be variable [5].

PTSD and ASD are trauma and stressor-related psychiatric disorders that could occur after experiencing or witnessing events involving physical injury, death, or other threats to the physical integrity [4]. Re-experience of the traumatic event and avoidance of trauma-related stimuli are the main symptoms of trauma and stressor-related disorders [4]. Unlike PTSD and ASD, depression is a mental disorder that is not directly linked to a traumatic event. However, substantial depression prevalence rates have been reported among patients who experienced a traumatic event such as interpersonal violence [6, 7]. A depressive episode is characterized by a constant depressed mood, loss of interest, or loss of pleasure [4].

A systematic review by Santiago et al. [5] reported that PTSD trajectories differ between patients exposed to intentional and non-intentional traumatic events. The PTSD prevalence among patients exposed to non-intentional traumatic events decreased in time, whereas the prevalence among patients exposed to intentional traumatic events increased. This might suggest that the PTSD trajectory of patients with violence-related injury differs from patients with non-intentional injury. However, Santiago et al. [5] also included studies on victims of terroristic attacks, war, and hostage situations in their systematic review. These participants did not necessarily sustained injury. It therefore remains unclear what the specific trajectories are for patients with violence related injury. Furthermore, little is known about the prevalence and trajectories of ASD and depression in this specific population.

In order to gain insight into the total health impact of injury following violence, the psychological consequences should be taken into account given the high prevalence rates of PTSD, ASD, and depression that have been reported post-injury [5, 8–10]. This paper provides PTSD, ASD, and depression prevalence estimates among patients with violence related injury, which may inform our understanding of both prognosis and recovery for these patients. An overview of prevalence rates provides insight into the public health treatment needs. Targeted interventions can be provided when the PTSD, ASD and depression trajectories of patients who sustained violence related injury are known. Therefore, we aim to (1) provide an overview of the published literature reporting the prevalence rates and trajectories of ASD, PTSD, and depression following violence related injury, and (2) to assess the quality of the studies included.

## Methods

In order to identify studies reporting the prevalence rates of ASD, PTSD, and depression among patients who sustained violence related injury, a systematic literature review was conducted. The methods and reporting of this systematic review are in concordance with the PRISMA statement on reporting standards for systematic reviews [11]. The study protocol is registered in the PROSPERO international prospective register of systematic reviews (registration number CRD42016043167).

### Literature search

Relevant studies were identified through systematic literature searches in the EMBASE, MEDLINE, Cochrane Central, PubMed, and PsycINFO databases. The search strategies were developed in consultation with a medical librarian. A detailed description of the search strategy can be found in the Additional file 1. Reference lists and citation indices of the included papers were inspected to identify additional relevant citations. We restricted searches to English-language papers, published in peer-reviewed journals before November 2017.

### Study selection

Studies reporting the prevalence of PTSD, ASD, or depression after ED or hospital treated injury following interpersonal violence were included in this review. We defined the following inclusion and exclusion criteria:

#### Participants

Studies were included if the injury was intentionally caused by another person or persons, such as (sexual) assault or stabbing. Studies on violent incidents that not necessarily involve injury, such as hostage situations or witnessing terroristic attacks, were excluded. Studies on a mixed population, e.g. all trauma patients, were only included if they reported separate prevalence rates for injury caused by intentional violence (excluding self-harm). We only included studies on patients who have been treated at the ED or hospital in order to maintain comparability in terms of injury severity. We did not apply restrictions on countries or regions in which studies were conducted. Studies on adults, children, and adolescents were included.

#### Outcome

We included studies in which the prevalence rates of PTSD, ASD or depression were reported directly or indirectly (i.e. by reporting the number of cases and the total number of patients) based on a validated questionnaire or diagnostic interview. We applied the case definitions and diagnostic thresholds as reported in the individual studies.

#### Study design

Prospective and retrospective cohort studies, longitudinal studies, cross-sectional studies, time series, and clinical trials were included. We excluded reviews, qualitative studies, case reports, editorials, and study protocols.

### Data extraction

Titles and abstracts of all identified studies were screened for relevance by one reviewer (RO, BO, or JH). After initial selection, the remaining records were independently read in full-text by two reviewers (RO and BO) for the eligibility assessment. Discrepancies were discussed and resolved by consulting a third reviewer (JH). Two reviewers (RO and BO) extracted data on the study populations, study setting, injury details, prevalence rates, diagnostic instruments, and follow-up. If possible, we provided prevalence rates at different points in time. We used approximations when specific time points were not reported. For example, when ‘within two weeks after ED admission’ was reported as time indication, the midpoint (one week) was used. We reported gender-specific prevalence rates and measures of injury severity if provided.

### Quality assessment

A quality assessment in terms of risk of bias was performed with the Quality in Prognosis Studies (QUIPS) tool [12], which was developed for assessing the risk of bias of prognostic studies. Although the current systematic review does not focus on prognostic studies, we used the QUIPS tool because it covers general quality criteria on risk of bias. We considered these general criteria as appropriate because of the variety of study designs included in our study. The following domains of the QUIPS were selected in order to assess the risk of bias: study participation, study attrition, outcome measurement, and statistical analysis. Two reviewers (RO and BO) independently used the QUIPS tool to assess the risk of bias. Each domain was scored as ‘low risk’, ‘moderate risk’ or ‘high risk’. Any discrepancies in the domain scores were resolved via discussion until consensus was reached.

## Results

### Literature search

**Fig. 1:**
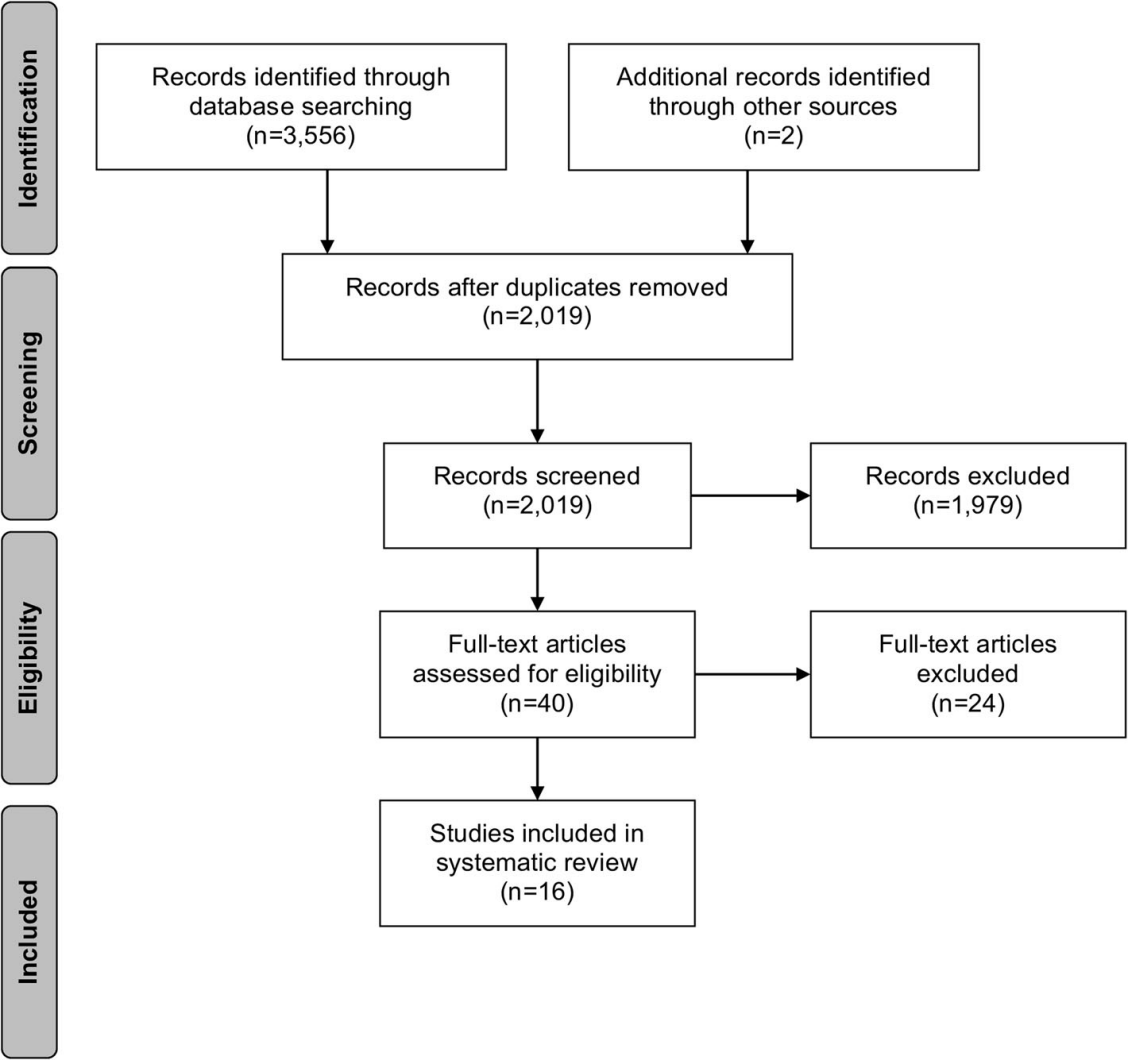
Flow chart of the study identification process

In total, the literature search yielded 3556 articles. After excluding 1537 duplicates, the titles and abstracts of 2019 articles were screened for relevance. The screening of titles and abstracts resulted in the exclusion of 1979 articles. Forty studies were left for full-text eligibility assessment, of which 24 were excluded for several main reasons: no prevalence reported, no violence related injury, no ED or hospital admission, literature review. Finally, sixteen studies were included in the systematic review. A flow chart of the study identification process is presented in Fig. 1.

### Study characteristics

The majority of the studies were conducted in the United States (*n* = 10) [13–22] (Table 1). The remaining studies were conducted in the United Kingdom (*n* = 3) [23–25], Denmark (*n* = 1) [26], and Norway (*n* = 2) [27, 28]. Seven studies included patients aged eighteen years and older [14, 20–22, 26–28] and two studies included patients aged sixteen years and older [23, 25]. In two studies [13, 24], the age of the participants was not specified. The remaining five studies applied different age criteria (Table 1) [15, 19].

**Table 1:** Overview of the study characteristics reporting the prevalence of ASD, PTSD, or depression following violence related injury

Authors, year, country, ref.	Study population	Setting details	Violence and injury details	Disorder	DSM criteria^a^
Alarcon et al., 2012, USA, [13]	Injured patients treated at the ED, age not specified	Urban level I trauma center	Assault	PTSD	No
Bisson et al., 2010, UK, [23]	Patients aged over 16 years, treated at the ED following physical assault	ED	Assault	PTSD	No
Boccelari et al., 2007, USA, [14]	Patients aged 18 years and older who are victims of violent crime treated at the ED, with and without hospitalization	Urban hospital	All types of violence, sexual assault excluded	Depression, ASD	No
Cunningham et al., 2015, USA, [15]	Patients aged between 14 and 24 years treated at the ED following assault	Urban public ED, high crime rates in region	Assault, sexual assault and child abuse excluded	Depression, PTSD	Yes, DSM-IV
Elklit et al., 2003, Denmark, [26]	Patients aged 18 years and older who are victims of physical assault, treated at the ED	ED	Assault, mean Injury Severity Score 1.47, two-third of the sample had head and face injuries	ASD, PTSD	No
Fein et al., 2002, USA, [16]	Patients aged between 12 and 24 years treated at the ED for intentional violence	Urban EDs	Assault/fights, child abuse and domestic violence excluded	ASD, PTSD	No
Hunt et al., 2016, USA, [20]	Injured trauma survivors aged 18 years and older, admitted to trauma center, 8.6% of the patients were victims of intentional stabbing	Two level I trauma centers	Stabbing	PTSD	Yes, DSM-V
Johansen et al., 2006, Norway, [27]	Patients aged over 18, treated at the ED following assault	ED	Assault, domestic violence excluded	PTSD	No
Johansen et al., 2007, Norway, [28]	Patients aged 18 years and older, treated at the ED following physical assault	ED	Assault, domestic violence excluded	PTSD	No
Kleim et al., 2007, UK, [24]	Patients treated at the ED following assault, mean age 35 years	ED	Assault, domestic violence excluded	ASD, PTSD	PTSD only, DSM-IV
McCart et al., 2005, USA, [17]	Patients aged 9–18 years, treated at the ED following assault	ED	Assault, with and without weapons	Depression, PTSD	No
Pailler et al., 2007, USA, [18]	Patients aged between 12 and 17, treated at the ED following a violence-related event	ED	Violent event, child abuse excluded	Depression, ASD, PTSD	No
Purtle et al., 2014, USA, [19]	Patients aged between 7 and 17 years who sustained intentional interpersonal injury treated at the ED	Urban level I trauma center	Violent event, child abuse, domestic violence, and sexual assault excluded	PTSD	No
Roy-Byrne et al., 2004, USA, [22]	Patients aged 18 years and older, admitted to ED following sexual or physical assault, not requiring hospitalization	Urban level I trauma center	Sexual or physical assault	PTSD	Yes, DSM-IV
Sullivan et al., 2017, USA, [21]	Patients aged 18 years and older, admitted to the trauma service for at least 24 h following aggravated assault	Urban level I trauma center	Aggravated assault and gunshot wounds	Depression, PTSD	No
Walters et al., 2007, UK, [25]	Patients aged over 16 years, treated at the ED following assault	ED	Assault, no further exclusion criteria	PTSD	No

*ASD* acute stress disorder, *ED* emergency department, *PTSD* post-traumatic stress disorder

^a^Are all DSM-IV or DSM-V diagnostic criteria for ASD, PTSD, or depression met, e.g. assessed by means of a structured clinical interview?

All studies included patients who presented to the ED, trauma center, or hospital with injury following intentional violence. Alarcon et al. [13] included patients with the ICD-9-CM injury codes 800–995, covering injury such as open wounds and fractures. Injury related to sexual assault was excluded in three studies [14, 15, 19] and injury caused by domestic violence was excluded in four studies [19, 24, 27, 28]. In four studies on children and adolescents, injury caused by child abuse was excluded [15, 16, 18, 19].

### Diagnostic instruments

A full structured clinical interview was used as diagnostic instrument in four out of sixteen studies [15, 20, 22, 24]. All DSM IV or V diagnostic criteria for PTSD (*n* = 4) and depression (*n* = 1) were met in these studies (Table 2). The Child and Adolescents Trauma Survey for assessing PTSD symptoms was used as diagnostic instrument in two studies [16, 18]. In both studies, patients were considered having PTSD when they scored 27 or higher. The Immediate Stress Response Checklist for ASD was used in the same studies [16, 18], although one of these studies did not report a cut-off score [18]. The diagnostic instruments used in the other studies were all different from each other. Twelve out of sixteen studies (75%) used brief questionnaires based on self-report or screening measures to obtain probable diagnoses. Therefore, these studies may have included individuals who would not have met the diagnostic criteria for ASD, PTSD, or depression if a full diagnostic interview would have been conducted. Brief questionnaires are mainly focused on symptoms whereas in a full diagnostic interview impairment is assessed as well.

**Table 2:** Overview of PTSD, ASD, and depression prevalence rates and diagnostic instruments

PTSD (*n* = 15)	Instrument, cut-off	No./total no.	Prevalence in % (95% CI)				
			< 1 month	1 month	3 months	6 months	12 months
Alarcon et al. [13]	PCL-C, ≥35	7/16	–	43.7 (11.4–76.2)	–	–	–
Bisson et al. [23]	TSQ, ≥6	338/3349	59.1 (52.8–65.4)	–	–	–	–
Cunningham et al. [15]	MINI, DSM-IV criteria	30/184	–	–	–	–	16.3 (10.5–22.1)
Elklit et al. [26]	HTQ, ≥3 on all scales	26/118	–	–	–	22.0 (13.5–30.5)	–
Fein et al. [16]	CATS, ≥27	4/96	–	–	5.8 (0.12–11.5)	–	–
Hunt et al. [20]	CAPS, DSM-V criteria	7/12	–	58.3 (15.1–100)	–	–	–
Johansen et al. [27]	PTSS-10^a,b^	46/138	–	33.3 (23.7–43.0)^c^	–	–	–
Johansen et al. [28]	PTSS-10^a,b^	20/70, 17/70, 19–70	–	28.6 (16.0–41.1)	24.3 (12.7–35.8)	–	27.1 (14.9–39.3)
Kleim et al. [24]	SCID, DSM-IV criteria	49/205	–	–	–	23.9 (17.2–30.6)	–
McCart et al. [17]	TSCC, NR	7/89	7.1 (1.85–12.4)	–	–	–	–
Pailler et al. [18]	CATS, ≥27	3/158	–	–	–	1.9 (3.8–12.7)	–
Purtle et al. [19]	CTSQ, ≥5	31/47	66.0 (42.7–89.2)	–	–	–	–
Roy-Byrne et al. [22]	CAPS, DSM-V criteria	14/23, 7/23	–	60.9 (29.0–92.8)	30.4 (7.9–53.0)	–	–
Sullivan et al. [21]	PC-PTSD, ≥3	33/87	37.9 (25.0–50.9)	–	–	–	–
Walters et al. [25]	DTS^d^	NR	–	11 (NR)	–	7.7 (NR)	–
ASD (*N* = 5)	Instrument, cut-off	No./total no.	Prevalence in % (95% CI)				
			< 1 week	1 week	> 1 week		
Boccelari et al. [14]	ASDS, > 36	221/541	–	40.9 (35.5–46.2)	–		
Elklit et al. [26]	HTQ, ≥2	47/196	24.0 (17.1–30.8)	–	–		
Fein et al. [16]	ISRC^e^	17/69	24.6 (12.9–36.3)	–	–		
Kleim et al. [24]	ASDS, NR	37/222	–	–	16.7 (11.3–22.0)		
Pailler et al. [18]	ISRC, NR	46/394	–	11.7 (8.3–15.0)	–		
Depression (*N* = 5)	Instrument, cut-off	No./total no.	Prevalence in % (95% CI)				
			< 1 month	≥ 1 month			
Boccelari et al. [14]	PHQ, NR	191/541	35.3 (30.3–40.3)	–			
Cunningham et al. [15]	MINI, DSM-IV criteria	31/184	–	16.8 (10.9–22.8)			
McCart et al. [17]	TSCC, NR	5/89	5.1 (0.6–9.6)	–			
Pailler et al. [18]	CDI-SF, > 65	12/394	3.0 (1.3–4.8)	–			
Sullivan et al. [21]	PHQ-8, ≥10	36/87	41.4 (27.9–54.9)	–			

*ASD* acute stress disorder, *ASDS* Acute Stress Disorder Scale, *CAPS* Clinician Administered PTSD Scale, *CATS* Child and Adolescents Trauma Survey, *CDI-SF* Children’s Depression Inventory Short Form, *CTSQ* Child Trauma Screening Questionnaire, *DTS* Davidson Trauma Scale, *HTQ* Harvard Trauma Questionnaire, *ISRC* Immediate Stress Response Checklist, *MINI* Mini International Neuropsychiatric Interview, *NR* not reported, *PC-PTSD* Primary Care PTSD, *PCL-C* PTSD Checklist-Civilian, *PHQ(− 8)* Patient Health Questionnaire (8), *PTSD* post-traumatic stress disorder, *PTSS-10* Post Traumatic Symptom Scale 10, *SCID* Structured Clinical Interview for DSM-IV, *TSCC* Trauma Symptom Checklist for Children, *TSQ* Trauma Screening Questionnaire

^a^Cut-off: a score of four or more on six or more items indicating PTSD

^b^IES-15 (Impact of Event Scale 15) was used as a secondary instrument, prevalence rates: 25.7% 1 month, 30.0% 3 months, 31.4% 12 months

^c^Males: 33/110 (30%), females: 13/28 (46%)

^d^Cut-off: at least one re-experiencing, three avoidance and two hyperarousal symptoms at a frequency of at least twice in the previous week

^e^Cut-off: at least one significant symptom in every category

### Prevalence rates

The PTSD, ASD, and depression prevalence rates at different points in time are reported in Table 2. Fifteen studies reported the prevalence of PTSD following violence related injury [13, 15–28], five studies reported the prevalence of ASD [14, 16, 18, 24, 26], and five studies reported the prevalence of depression [14, 15, 17, 18, 21]. The PTSD prevalence at 1, 3, 6, and 12 months post-injury ranged between 11.0–60.9%, 5.8–30.4%, 1.9–23.9%, and 16.3–27.1% respectively. The following range of ASD prevalence rates were reported < 1 week post-injury and 1–2 weeks post-injury: 24.0–24.6% and 11.7–40.6%. Four studies reported depression prevalence rates < 1 month post-injury ranging between 3.0 and 35.3%. Beyond one month post-injury, a prevalence rate of 16.8% was reported. Heterogeneity resulting from the use of different diagnostic instruments strongly limited the comparability of the reported prevalence rates of PTSD, ASD, and depression. In total, one study reported injury severity of the target population [26] and one study reported gender-specific prevalence rates [27] (Table 2).

### Quality assessment

Of all 64 possible scoring options (four quality domains times sixteen studies), the reviewers disagreed on five scoring options resulting in a disagreement rate of 7.8%. Two of the disagreements belonged to the study participation domain and three to the outcome measurement domain. Disagreements were resolved after discussion. Table 3 describes the risk of bias per domain (study participation, study attrition, outcome measurement, and statistical analysis) for all studies included. The study by Pailler et al. [18] was the only study with a low risk of bias on all four domains. The study attrition domain was mainly scored as high risk (83%) because of low participation rates and/or poor descriptions of the patients lost to follow-up. One study scored ‘low risk’ in this domain [18]. The statistical analyses and the presentation of the results were adequate in all studies. Therefore, all studies scored ‘low risk’ on the statistical analyses domain. The outcome measurement domain was mainly scored as low risk (67%). The majority had a low risk score for the study participation domain (67%), but one study had a high risk of bias because the recruitment process, inclusion criteria, and baseline characteristics were not reported adequately [26].

**Table 3:** QUIPS risk of bias assessment

Study	Study participation	Study attrition	Outcome measurement	Statistical analysis and presentation
Alarcon et al. [13]	Low	High	Low	Low
Bisson et al. [23]	Moderate	High	Low	NA
Boccelari et al. [14]	Moderate	High	Moderate	Low
Cunningham et al. [15]	Low	High	Moderate	Low
Elklit et al. [26]	High	High	Low	Low
Fein et al. [16]	Low	Moderate	Moderate	Low
Hunt et al. [20]	Low	Moderate	Low	Low
Johansen et al. [27]	Low	High	Moderate	Low
Johansen et al. [28]	Low	High	Moderate	Low
Kleim et al. [24]	Low	High	Low	Low
McCart et al. [17]	Moderate	High	Low	Low
Pailler et al. [18]	Low	Low	Low	Low
Purtle et al. [19]	Low	High	Low	Low
Roy-Byrne et al. [22]	Low	High	Low	Low
Sullivan et al. [21]	Low	High	Low	Low
Walters et al. [25]	Low	High	Low	Low

*NA* not applicable

## Discussion

This systematic review provides an overview of the published literature reporting the prevalence rates and trajectories of PTSD, ASD, and depression following violence related injury treated at the ED or hospital. The quality of the included studies was assessed. We identified sixteen studies reporting the prevalence of ASD, PTSD, or depression. The reported prevalence rates were diverse across different follow-up points resulting in a wide range. The quality assessment indicated that almost all studies were susceptible to bias due to low response rates and loss to follow-up.

In a previous meta-analysis on the prevalence of PTSD among trauma-exposed children and adolescents, an overall pooled prevalence rate of 15.9% was reported [29]. The pooled prevalence rate for victims of interpersonal violence was 25.2%. The time of diagnosis was not specified, however. We found prevalence rates ranging from 1.9% (3 months) to 66% (< 1 month) among children and adolescents. It is not warranted to aggregate these prevalence rates given the differences in the timing of the diagnosis and diagnostic instruments. White et al. [30] reported a PTSD prevalence of 14.3% among an adult sample that experienced a traumatic event. Again, this finding is difficult to compare with our results as the PTSD prevalence ranged from 7.7% (6 months) to 60.9% (< 1 month). Brewin et al. [31] reported an ASD prevalence estimate of 19% among adult violent crime victims who were not necessarily treated for injury. This prevalence rate is comparable with the ASD prevalence rates reported in four included studies (11.7–24.6%), but one study reported a prevalence rate of 41% [14]. These findings suggest that ASD is highly prevalent in patients with violence related injury and that the prevalence is comparable to populations consisting of injured and non-injured violence victims.

Four studies reported PTSD prevalence rates before one month after the traumatic event [17, 19, 21, 23], which is not in accordance with the DSM (IV and V) criteria. It could be possible that these PTSD symptoms resulted from other traumatic events. Data on pre-existing PTSD, ASD, and depression among the study samples were not available, however. Consequently, it is unclear whether mental disorders were already present prior to the injury. This limitation is common in violence and injury research, but has to be taken into account when interpreting the results. It is also possible that people who already have PTSD, ASD or depression are more likely to be involved in interpersonal violence. It is known that PTSD is associated with more risk behavior [32] which could increase the likelihood of involvement in violence. Information regarding the diagnostic status before the injury is therefore valuable for interpreting the prevalence rates.

All studies were conducted in high-income countries, of which the vast majority in the United States. The findings of this review are therefore limited to these countries. Health care systems in high-income countries are relatively well established, which facilitates recognition, prevention, and treatment. It is therefore likely that the prevalence rates and trajectories of PTSD, ASD, and depression are different in middle and low-income countries.

### Strengths and limitations

One of the strengths of our study is that standard methods for conducting and reporting systematic reviews were followed [11]. Furthermore, psychological, medical, and other relevant literature databases were searched exhaustively. Another strength is that we assessed the quality of the included studies. A limitation of our review is that the search was restricted to studies published in scientific peer-reviewed journals in English language. We did not consider dissertations, unpublished material or studies in non-English language, which could have biased our findings.

### Recommendations

For future research, we recommend uniformity in diagnostic procedures. Structured diagnostic interviews by a clinician are preferred, but this is often not feasible. These interviews are time consuming and costly as they require involvement of trained professionals. Nevertheless, validated questionnaires can be used as an approximation. Our findings show that a large variety of questionnaires are available, however. Estimates of PTSD prevalence tend to vary according to the diagnostic criteria used, which underpins the need for uniformity in diagnostic procedures. These differences in diagnostic procedures could be reduced by establishing international guidelines on assessing mental health problems among trauma patients. Although international uniformity in diagnostic procedures would increase the comparability of PTSD, ASD and depression estimates, one should pay attention to ethnocultural differences. The validity of responses to measures may vary between populations, cultures, and countries [33]. Values and norms associated with culture guide perception and individual responses, including psychiatric symptoms [34]. Marshall et al. [35] investigated posttraumatic stress among a sample of Hispanic, non-Hispanic Caucasian, and African American survivors of physical injury. They found that the Hispanic group reported different symptoms and higher levels of overall posttraumatic distress. Such results raise questions regarding whether certain cultures truly experience higher levels of distress after experiencing a traumatic event, or whether cultural factors have an impact on the symptom manifestation only.

One of the sixteen included studies reported gender-specific prevalence rates. We recommend to report gender specific prevalence rates, since it is known that women are more likely to develop PTSD after trauma than men [29, 36]. Trajectories of PTSD, ASD, and depression can be better understood when distinguishing gender specific prevalence rates.

Prevalence rates should also be reported separately for injury types, such as sexual versus physical assault injuries and injuries caused by strangers versus family. The studies in the current review included patients with different injury types but prevalence rates were not reported separately. Identifying injury types that are associated with higher rates of PTSD, ASD, or depression may lead to earlier identification of high risk patients. Furthermore, ethnocultural differences in prevalence estimates should be considered in future studies. Cultural factors shape the subjective meaning of traumatic events, which in turn influences symptom expression [37].

Only few studies had follow-up measurements beyond one year after the violent incident. Previous studies suggest that the course of PTSD may vary over time. Prospective assessments are required to study the course of mental disorders following violence related injury treated at the ED or hospital. Since there are indications that the prevalence of PTSD among victims of intentional violence increases over time [5] it is relevant to know what the trajectories of PTSD and other mental disorders are for individuals who sustained injury following violence. For future research, extending the follow-up could contribute to better understanding of mental disorder trajectories following violence related injury.

## Conclusions

Heterogeneity resulting from the use different diagnostic instruments limited the comparability of the ASD, PTSD, and depression prevalence rates. The reported prevalence rates should be interpreted carefully as almost all studies were susceptible to bias due to low response rates. Definitive or broad statements on the prevalence rates and trajectories are therefore not warranted. Study participation and loss to follow-up require more attention in future studies. Uniformity in diagnostic procedures is needed for future studies on mental disorders following violence related injury.

## Additional file

Additional file 1: Search strategy. (DOCX 19 kb)

## Abbreviations

ASD: acute stress disorder
DSM: Diagnostic and Statistical Manual of Mental Disorders
ED: emergency department
PTSD: post-traumatic stress disorder
QUIPS: Quality in Prognosis Studies
