# Supplementary material for: Prevalence of post-traumatic stress disorder, acute stress disorder and depression following violence related injury treated at the emergency department: a systematic review
Source: BMC Psychiatry. 2018 Sep 25;18:311. doi: 10.1186/s12888-018-1890-9 (PMC6156976; doi:10.1186/s12888-018-1890-9)
Supplement: Supplementary file 1 — Search strategy. (DOCX 19 kb) [file 12888_2018_1890_MOESM1_ESM.docx]

**Additional file 1: Search strategy**

**Embase.com**

('mental stress'/de OR 'acute stress disorder'/exp OR depression/exp OR 'posttraumatic stress disorder'/exp OR 'anxiety disorder'/exp OR 'mental disease'/exp OR 'mental health'/exp OR 'Diagnostic and Statistical Manual of Mental Disorders'/exp OR (((stress OR anxi* OR dystress* OR panic OR mental* OR psych*) NEAR/3 (disorder* OR trauma* OR posttrauma* OR ill* OR consequen* OR profile* OR comorbid* OR reaction*)) OR (psych* NEAR/3 (problem* OR burden*)) OR depress* OR dysthymi* OR ptsd OR 'mental health' OR dsm):ab,ti) AND (violence/exp OR victim/exp OR crime/de OR 'gunshot injury'/exp OR 'stab wound'/exp OR 'blunt trauma'/exp OR (violen* OR assault* OR ((physical* OR domestic*) NEAR/3 (abuse* OR attack*)) OR battered* OR battering* OR victim* OR crime OR crimes OR ciminal* OR stabbing* OR stab OR ((blunt OR sharp) NEAR/3 (object* OR trauma*)) OR fight* OR beating OR beaten OR beated OR (intention* NEAR/3 (injur* OR hurt*)) OR ballistic* OR gunshot OR bullet* OR firearm* OR gun OR guns OR shotgun):ab,ti) AND (emergency/de OR 'emergency ward'/exp OR 'emergency care'/exp OR 'emergency health service'/exp OR 'emergency medicine'/exp OR 'emergency patient'/exp OR 'emergency treatment'/de OR 'emergency nursing'/exp OR (emergenc* OR (trauma NEAR/3 (centre* OR center* OR service*)) OR er OR ers OR ((ed OR eds) NOT (eating NEXT/1 disorder*))):ab,ti) AND (prevalence/exp OR incidence/de OR 'correlation analysis'/exp OR 'correlation coefficient'/exp OR 'disease association'/exp OR screening/de OR (prevalen* OR incidence* OR correlate* OR (co NEXT/1 occurr*) OR screen*):ab,ti)

**Medline (OvidSP)**

(exp Mental Disorders/ OR "Stress, Psychological"/ OR depression/ OR exp "Depressive Disorder"/ OR exp mental health/ OR Diagnostic and Statistical Manual of Mental Disorders/ OR (((stress OR anxi* OR dystress* OR panic OR mental* OR psych*) ADJ3 (disorder* OR trauma* OR posttrauma* OR ill* OR consequen* OR profile* OR comorbid* OR reaction*)) OR (psych* ADJ3 (problem* OR burden*)) OR depress* OR dysthymi* OR ptsd OR mental health OR dsm).ab,ti.) AND (exp violence/ OR Crime Victims/ OR exp crime/ OR Wounds, Gunshot/ OR Wounds, Stab/ OR (violen* OR assault* OR ((physical* OR domestic*) ADJ3 (abuse* OR attack*)) OR battered* OR battering* OR victim* OR crime OR crimes OR ciminal* OR stabbing* OR stab OR ((blunt OR sharp) ADJ3 (object* OR trauma*)) OR fight* OR beating OR beaten OR beated OR (intention* ADJ3 (injur* OR hurt*)) OR ballistic* OR gunshot OR bullet* OR firearm* OR gun OR guns OR shotgun).ab,ti.) AND (exp emergencies/ OR exp Emergency Service, Hospital/ OR Emergency Medical Services/ OR Emergency Medicine/ OR exp Emergency Treatment/ OR exp Emergency Nursing/ OR (emergenc* OR (trauma ADJ3 (centre* OR center* OR service*)) OR er OR ers OR ((ed OR eds) NOT (eating ADJ disorder*))).ab,ti.) AND (prevalence/ OR incidence/ OR correlation analysis/ OR correlation coefficient/ OR disease association/ OR screening/ OR (prevalen* OR incidence* OR correlate* OR (co ADJ occurr*) OR screen*).ab,ti.)

**PsycINFO (OvidSP)**

(exp Mental Disorders/ OR "Stress"/ OR exp major depression/ OR "Depression (Emotion)"/ OR exp mental health/ OR "Diagnostic and Statistical Manual"/ OR (((stress OR anxi* OR dystress* OR panic OR mental* OR psych*) ADJ3 (disorder* OR trauma* OR posttrauma* OR ill* OR consequen* OR profile* OR comorbid* OR reaction*)) OR (psych* ADJ3 (problem* OR burden*)) OR depress* OR dysthymi* OR ptsd OR mental health OR dsm).ab,ti.) AND (exp violence/ OR Crime Victims/ OR exp crime/ OR (violen* OR assault* OR ((physical* OR domestic*) ADJ3 (abuse* OR attack*)) OR battered* OR battering* OR victim* OR crime OR crimes OR ciminal* OR stabbing* OR stab OR ((blunt OR sharp) ADJ3 (object* OR trauma*)) OR fight* OR beating OR beaten OR beated OR (intention* ADJ3 (injur* OR hurt*)) OR ballistic* OR gunshot OR bullet* OR firearm* OR gun OR guns OR shotgun).ab,ti.) AND (Emergency Services/ OR Emergency Management/ OR (emergenc* OR (trauma ADJ3 (centre* OR center* OR service*)) OR er OR ers OR ((ed OR eds) NOT (eating ADJ disorder*))).ab,ti.) AND (Statistical Correlation/ OR screening/ OR (prevalen* OR incidence* OR correlate* OR (co ADJ occurr*) OR screen*).ab,ti.)

**Cochrane**

((((stress OR anxi* OR dystress* OR panic OR mental* OR psych*) NEAR/3 (disorder* OR trauma* OR posttrauma* OR ill* OR consequen* OR profile* OR comorbid* OR reaction*)) OR (psych* NEAR/3 (problem* OR burden*)) OR depress* OR dysthymi* OR ptsd OR 'mental health' OR

dsm):ab,ti) AND ((violen* OR assault* OR ((physical* OR domestic*) NEAR/3 (abuse* OR attack*)) OR battered* OR battering* OR victim* OR crime OR crimes OR ciminal* OR stabbing* OR stab OR ((blunt OR sharp) NEAR/3 (object* OR trauma*)) OR fight* OR beating OR beaten OR beated OR (intention* NEAR/3 (injur* OR hurt*)) OR ballistic* OR gunshot OR bullet* OR firearm* OR gun OR guns OR shotgun):ab,ti) AND ((emergenc* OR (trauma NEAR/3 (centre* OR center* OR service*)) OR er OR ers OR ((ed OR eds) NOT (eating NEXT/1 disorder*))):ab,ti) AND ((prevalen* OR incidence* OR correlate* OR (co NEXT/1 occurr*) OR screen*):ab,ti)

**Web-of-science**

TS=(((((stress OR anxi* OR dystress* OR panic OR mental* OR psych*) NEAR/3 (disorder* OR trauma* OR posttrauma* OR ill* OR consequen* OR profile* OR comorbid* OR reaction*)) OR (psych* NEAR/3 (problem* OR burden*)) OR depress* OR dysthymi* OR ptsd OR "mental health" OR dsm)) AND ((violen* OR assault* OR ((physical* OR domestic*) NEAR/3 (abuse* OR attack*)) OR battered* OR battering* OR victim* OR crime OR crimes OR ciminal* OR stabbing* OR stab OR ((blunt OR sharp) NEAR/3 (object* OR trauma*)) OR fight* OR beating OR beaten OR beated OR (intention* NEAR/3 (injur* OR hurt*)) OR ballistic* OR gunshot OR bullet* OR firearm* OR gun OR guns OR shotgun)) AND ((emergenc* OR (trauma NEAR/3 (centre* OR center* OR service*)) OR er OR ers OR ((ed OR eds) NOT (eating NEAR/1 disorder*)))) AND ((prevalen* OR incidence* OR correlate* OR (co NEAR/1 occurr*) OR screen*)))

**Scopus**

TITLE-ABS-KEY(((((stress OR anxi* OR dystress* OR panic OR mental* OR psych*) W/3 (disorder* OR trauma* OR posttrauma* OR ill* OR consequen* OR profile* OR comorbid* OR reaction*)) OR (psych* W/3 (problem* OR burden*)) OR depress* OR dysthymi* OR ptsd OR "mental health" OR dsm)) AND ((violen* OR assault* OR ((physical* OR domestic*) W/3 (abuse* OR attack*)) OR battered* OR battering* OR victim* OR crime OR crimes OR ciminal* OR stabbing* OR stab OR ((blunt OR sharp) W/3 (object* OR trauma*)) OR fight* OR beating OR beaten OR beated OR (intention* W/3 (injur* OR hurt*)) OR ballistic* OR gunshot OR bullet* OR firearm* OR gun OR guns OR shotgun)) AND ((emergenc* OR (trauma W/3 (centre* OR center* OR service*)) OR er OR ers OR ((ed OR eds) AND NOT (eating W/1 disorder*)))) AND ((prevalen* OR incidence* OR correlate* OR (co W/1 occurr*) OR screen*)))

**PubMed publisher**

(Mental Disorders[mh] OR "Stress, Psychological"[mh] OR depression[mh] OR "Depressive Disorder"[mh] OR mental health[mh] OR Diagnostic and Statistical Manual of Mental Disorders[mh] OR (((stress OR anxi*[tiab] OR dystress*[tiab] OR panic OR mental*[tiab] OR psych*[tiab]) AND (disorder*[tiab] OR trauma*[tiab] OR posttrauma*[tiab] OR ill*[tiab] OR consequen*[tiab] OR profile*[tiab] OR comorbid*[tiab] OR reaction*[tiab])) OR (psych*[tiab] AND (problem*[tiab] OR burden*[tiab])) OR depress*[tiab] OR dysthymi*[tiab] OR ptsd OR mental health OR dsm)) AND (violence[mh] OR Crime Victims[mh] OR crime[mh] OR Wounds, Gunshot[mh] OR Wounds, Stab[mh] OR (violen*[tiab] OR assault*[tiab] OR ((physical*[tiab] OR domestic*[tiab]) AND (abuse*[tiab] OR attack*[tiab])) OR battered*[tiab] OR battering*[tiab] OR victim*[tiab] OR crime OR crimes OR ciminal*[tiab] OR stabbing*[tiab] OR stab OR ((blunt OR sharp) AND (object*[tiab] OR trauma*[tiab])) OR fight*[tiab] OR beating OR beaten OR beated OR (intention*[tiab] AND (injur*[tiab] OR hurt*[tiab])) OR ballistic*[tiab] OR gunshot OR bullet*[tiab] OR firearm*[tiab] OR gun OR guns OR shotgun)) AND (emergencies[mh] OR Emergency Service, Hospital[mh] OR Emergency Medical Services[mh] OR Emergency Medicine[mh] OR Emergency Treatment[mh] OR Emergency Nursing[mh] OR (emergenc*[tiab] OR (trauma AND (centre*[tiab] OR center*[tiab] OR service*[tiab])) OR er OR ers OR ((ed OR eds) NOT eating disorder*[tiab]))) AND (prevalence[mh] OR incidence[mh] OR correlation analysis[mh] OR correlation coefficient[mh] OR disease association[mh] OR screening[mh] OR (prevalen*[tiab] OR incidence*[tiab] OR correlate*[tiab] OR co occurr*[tiab] OR screen*[tiab])) AND publisher[sb]
